# 3-Methyl-4-[(*E*)-3-thien­ylmethyl­idene­amino]-1*H*-1,2,4-triazole-5(4*H*)-thione

**DOI:** 10.1107/S1600536810041152

**Published:** 2010-10-20

**Authors:** Mohammad Asad, Chuan-Wei Oo, Hasnah Osman, Chin Sing Yeap, Hoong-Kun Fun

**Affiliations:** a School of Chemical Sciences, Universiti Sains Malaysia, 11800 USM, Penang, Malaysia; b X-ray Crystallography Unit, School of Physics, Universiti Sains Malaysia, 11800 USM, Penang, Malaysia

## Abstract

The asymmetric unit of the title compound, C_8_H_8_N_4_S_2_, contains two crystallographically independent mol­ecules. The thio­phene ring of one mol­ecule is disordered over two positions with refined site occupancies of 0.6375 (19) and 0.3625 (19). One mol­ecule is almost planar and the other one is twisted, the dihedral angles between the thio­phene and triazole rings being 7.28 (7) and 48.9 (2)° [48.5 (4)° for the minor component], respectively. An intra­molecular C—H⋯S hydrogen bond stabilizes the mol­ecular conformation of the planar molecule. In the crystal, the two mol­ecules are inter­connected by N—H⋯S hydrogen bonds into dimers, which are further consolidated into chains along the *b* axis by C—H⋯N hydrogen bonds. Weak C–H⋯π and π–π inter­actions [centroid–centroid distance = 3.5149 (7) Å] are also observed.

## Related literature

For general background and the biological activity of Schiff bases of 1,2,4-triazole derivatives, see: Ghazzali *et al.* (2010 ▶); Xia *et al.* (2010 ▶); Aytac *et al.* (2009 ▶); Siddiqui *et al.* (2006 ▶); Kucukguzel *et al.* (2008 ▶). For the stability of the temperature controller used in the data collection, see: Cosier & Glazer (1986 ▶).
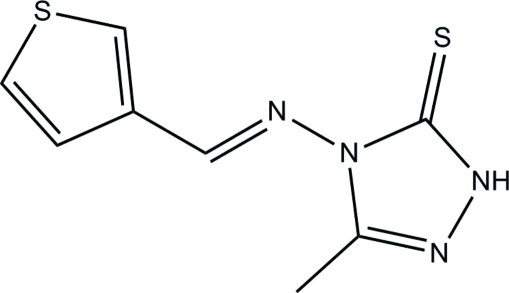


## Experimental

### 

#### Crystal data

C_8_H_8_N_4_S_2_
*M* *_r_* = 224.30
Triclinic, 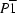

*a* = 9.3108 (7) Å
*b* = 10.2848 (8) Å
*c* = 12.7798 (10) Å
α = 66.632 (2)°
β = 83.409 (2)°
γ = 63.974 (2)°
*V* = 1006.88 (13) Å^3^
*Z* = 4
Mo *K*α radiation
μ = 0.49 mm^−1^
*T* = 100 K
0.36 × 0.25 × 0.23 mm

#### Data collection

Bruker APEXII DUO CCD area-detector diffractometer
Absorption correction: multi-scan (*SADABS*; Bruker, 2009 ▶) *T*_min_ = 0.845, *T*_max_ = 0.896
23519 measured reflections
8745 independent reflections
7500 reflections with *I* > 2σ(*I*)
*R*_int_ = 0.025

#### Refinement

*R*[*F* ^2^ > 2σ(*F* ^2^)] = 0.040
*wR*(*F* ^2^) = 0.114
*S* = 1.06
8745 reflections
288 parameters
1 restraint
H atoms treated by a mixture of independent and constrained refinement
Δρ_max_ = 1.06 e Å^−3^
Δρ_min_ = −0.50 e Å^−3^

### 

Data collection: *APEX2* (Bruker, 2009 ▶); cell refinement: *SAINT* (Bruker, 2009 ▶); data reduction: *SAINT*; program(s) used to solve structure: *SHELXTL* (Sheldrick, 2008 ▶); program(s) used to refine structure: *SHELXTL*; molecular graphics: *SHELXTL*; software used to prepare material for publication: *SHELXTL* and *PLATON* (Spek, 2009 ▶).

## Supplementary Material

Crystal structure: contains datablocks global, I. DOI: 10.1107/S1600536810041152/rz2500sup1.cif

Structure factors: contains datablocks I. DOI: 10.1107/S1600536810041152/rz2500Isup2.hkl

Additional supplementary materials:  crystallographic information; 3D view; checkCIF report

## Figures and Tables

**Table 1:** Hydrogen-bond geometry (Å, °)

*D*—H⋯*A*	*D*—H	H⋯*A*	*D*⋯*A*	*D*—H⋯*A*
N3*A*—H3*NA*⋯S2*B*^i^	0.894 (19)	2.46 (2)	3.3494 (11)	177.7 (18)
N3*B*—H3*NB*⋯S2*A*^ii^	0.84 (2)	2.45 (2)	3.2728 (12)	167.7 (19)
C5*A*—H5*AA*⋯S2*A*	0.93	2.50	3.2311 (12)	135
C8*B*—H8*BA*⋯N4*A*^iii^	0.96	2.59	3.5503 (16)	175
C5*B*—H5*BA*⋯*Cg*1^iv^	0.93	2.91	3.4955 (12)	122

Symmetry codes: (i) 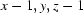; (ii) 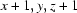; (iii) 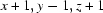; (iv) 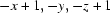.

**Fig. 1.**
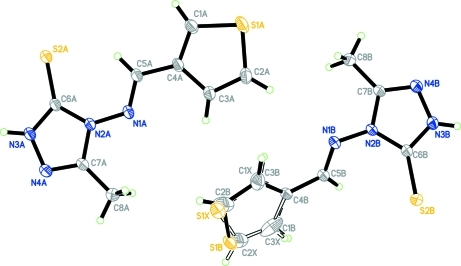
The molecular structure of the title compound with atom labels and 50% probability ellipsoids for non-H atoms.

**Fig. 2.**
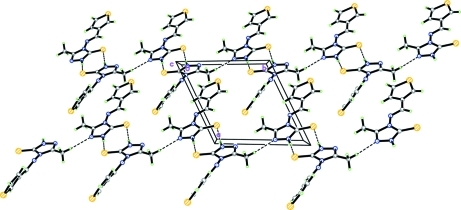
The crystal packing of the title compound viewed down the c axis showing chains along the b axis. Only the major component of disorder is shown. Intermolecular hydrogen bonds are shown as dashed lines.

## supplementary crystallographic information

### Comment

Schiff bases of 1,2,4-triazole and its derivatives have been the subject of current research in the field of pharmacology and coordination chemistry (Ghazzali *et al.*, 2010). Due to the bioactivity associated with substituted 1,2,4-triazoles, researchers and chemists are very much interested to study the chemistry of these compounds, as they exhibit a broad spectrum of biological properties such as anticancer (Xia *et al.*, 2010), anti-inflammatory/analgesic (Aytac *et al.*, 2009), antibacterial/antifungal (Siddiqui *et al.*, 2006), antiviral/anti-HIV and anti-tuberculosis (Kucukguzel *et al.*, 2008) activities.

The asymmetric unit of the title compound consists of two crystallographically independent molecules (Fig. 1). The thiophene ring of molecule *B* is disordered over two positions with refined site occupancies of 0.6375 (19) and 0.3625 (19). Both molecules exist in an *E* configuration with respect to the central C=N double bond. Molecule *A* is almost planar and molecule *B* is twisted, the dihedral angles between the thiophene ring and the triazole ring being 7.28 (7)° and 48.9 (2)° [48.5 (4)° for the minor component] respectively. Intramolecular C—H···S hydrogen bonds stabilize the molecular structures. In the crystal structure, the two molecules are interconnected by N3A—H3NA···S2B and N3B—H3NB···S2A hydrogen bonds (Table 1) into dimers and these dimers are further consolidated into chains along the *b* axis (Fig. 2) by C8B—H8BA···N4A hydrogen bonds (Table 1). Weak C–H···π and π···π interactions are also observed [*Cg*1···*Cg*2^v^ = 3.5149 (7) Å; (v) 1 - *x*, -*y*, -*z. Cg*1 and *Cg*2 are centroids of S1A–C1A–C4A–C3A–C2A and N2A–C6A–N3A–N4A–C7A ring, respectively].

### Experimental

A mixture of 3-methyl-4-amino-5-mercapto-1,2,4-triazole (4.46 mmol, 0.58 g) and thiophene-3-carboxaldehyde (4.46 mmol, 0.5 g) containing pyridine (0.1 ml) in ethanol was refluxed for about 13 to 14 h. The reaction mixture was cooled to room temperature and the light yellow solid was filtered off, washed with water, dried and recrystallized from chloroform-methanol (1:1 *v*/*v*) to get the title compound in 65% yield.

### Refinement

The thiophene ring of molecule *B* is disordered over two positions with refined site occupancies of 0.6375 (19) and 0.3625 (19). The same *U*_ij_ parameters were used for the atom pairs C1B/C1X and C2B/C2X. The S1X–C2X bond distance was constrained to 1.70 (1) Å. The N-bound hydrogen atoms was located in a difference Fourier map and refined freely. The rest of hydrogen atoms were positioned geometrically [C–H = 0.93–0.96 Å] and refined using a riding model [*U*_iso_(H) = 1.2–1.5*U*_eq_(C)]. A rotating-group model were applied for methyl groups.

### Crystal data

**Table:**

C_8_H_8_N_4_S_2_	*Z* = 4
*M**_r_* = 224.30	*F*(000) = 464
Triclinic, *P*1	*D*_x_ = 1.480 Mg m^−^^3^
Hall symbol: -P 1	Mo *K*α radiation, λ = 0.71073 Å
*a* = 9.3108 (7) Å	Cell parameters from 9994 reflections
*b* = 10.2848 (8) Å	θ = 2.4–35.0°
*c* = 12.7798 (10) Å	µ = 0.49 mm^−^^1^
α = 66.632 (2)°	*T* = 100 K
β = 83.409 (2)°	Block, colourless
γ = 63.974 (2)°	0.36 × 0.25 × 0.23 mm
*V* = 1006.88 (13) Å^3^	

### Data collection

**Table:**

Bruker APEXII DUO CCD area-detector diffractometer	8745 independent reflections
Radiation source: fine-focus sealed tube	7500 reflections with *I* > 2σ(*I*)
graphite	*R*_int_ = 0.025
φ and ω scans	θ_max_ = 35.1°, θ_min_ = 2.4°
Absorption correction: multi-scan (*SADABS*; Bruker, 2009)	*h* = −15→13
*T*_min_ = 0.845, *T*_max_ = 0.896	*k* = −16→16
23519 measured reflections	*l* = −17→20

### Refinement

**Table:**

Refinement on *F*^2^	Primary atom site location: structure-invariant direct methods
Least-squares matrix: full	Secondary atom site location: difference Fourier map
*R*[*F*^2^ > 2σ(*F*^2^)] = 0.040	Hydrogen site location: inferred from neighbouring sites
*wR*(*F*^2^) = 0.114	H atoms treated by a mixture of independent and constrained refinement
*S* = 1.06	*w* = 1/[σ^2^(*F*_o_^2^) + (0.0606*P*)^2^ + 0.2963*P*] where *P* = (*F*_o_^2^ + 2*F*_c_^2^)/3
8745 reflections	(Δ/σ)_max_ = 0.001
288 parameters	Δρ_max_ = 1.06 e Å^−^^3^
1 restraint	Δρ_min_ = −0.50 e Å^−^^3^

### Special details

**Table:**

Experimental. The crystal was placed in the cold stream of an Oxford Cryosystems Cobra open-flow nitrogen cryostat (Cosier & Glazer, 1986) operating at 100.0 (1) K.
Geometry. All e.s.d.'s (except the e.s.d. in the dihedral angle between two l.s. planes) are estimated using the full covariance matrix. The cell e.s.d.'s are taken into account individually in the estimation of e.s.d.'s in distances, angles and torsion angles; correlations between e.s.d.'s in cell parameters are only used when they are defined by crystal symmetry. An approximate (isotropic) treatment of cell e.s.d.'s is used for estimating e.s.d.'s involving l.s. planes.
Refinement. Refinement of *F*^2^ against ALL reflections. The weighted *R*-factor *wR* and goodness of fit *S* are based on *F*^2^, conventional *R*-factors *R* are based on *F*, with *F* set to zero for negative *F*^2^. The threshold expression of *F*^2^ > σ(*F*^2^) is used only for calculating *R*-factors(gt) *etc*. and is not relevant to the choice of reflections for refinement. *R*-factors based on *F*^2^ are statistically about twice as large as those based on *F*, and *R*- factors based on ALL data will be even larger.

### Fractional atomic coordinates and isotropic or equivalent isotropic displacement parameters (Å^2^)

**Table:**

	*x*	*y*	*z*	*U*_iso_*/*U*_eq_	Occ. (<1)
S1A	0.76095 (4)	−0.34744 (4)	0.39899 (2)	0.02948 (7)	
S2A	0.22259 (3)	−0.11508 (3)	−0.03400 (2)	0.02363 (6)	
N1A	0.35008 (11)	0.05807 (10)	0.09471 (7)	0.02091 (15)	
N2A	0.24129 (11)	0.12778 (10)	0.00194 (7)	0.01941 (14)	
N3A	0.07864 (12)	0.20250 (10)	−0.13672 (8)	0.02245 (16)	
N4A	0.06835 (12)	0.33875 (11)	−0.13366 (8)	0.02392 (17)	
C1A	0.63594 (14)	−0.31400 (13)	0.29387 (9)	0.02447 (19)	
H1AA	0.6348	−0.3924	0.2755	0.029*	
C2A	0.68112 (15)	−0.15163 (15)	0.37468 (10)	0.0279 (2)	
H2AA	0.7137	−0.1108	0.4157	0.033*	
C3A	0.56310 (14)	−0.06294 (13)	0.28710 (9)	0.02428 (19)	
H3AA	0.5065	0.0456	0.2614	0.029*	
C4A	0.53698 (12)	−0.15641 (12)	0.23970 (8)	0.02067 (16)	
C5A	0.42312 (13)	−0.09238 (12)	0.14318 (8)	0.02136 (17)	
H5AA	0.4042	−0.1571	0.1179	0.026*	
C6A	0.18194 (12)	0.07072 (11)	−0.05621 (8)	0.01945 (16)	
C7A	0.16892 (13)	0.29067 (12)	−0.04888 (8)	0.02199 (17)	
C8A	0.20936 (16)	0.39193 (13)	−0.01317 (10)	0.0285 (2)	
H8AA	0.1551	0.4992	−0.0654	0.043*	
H8AB	0.3230	0.3597	−0.0134	0.043*	
H8AC	0.1762	0.3825	0.0624	0.043*	
S2B	0.88542 (4)	0.23323 (3)	0.64301 (2)	0.02461 (7)	
N1B	0.78425 (11)	0.05827 (10)	0.51301 (7)	0.02158 (16)	
N2B	0.85328 (11)	−0.01306 (10)	0.62466 (7)	0.01962 (15)	
N3B	0.97744 (12)	−0.07490 (11)	0.77934 (8)	0.02242 (16)	
N4B	0.98117 (13)	−0.21239 (11)	0.78403 (8)	0.02472 (17)	
C4B	0.58641 (12)	0.28766 (11)	0.37606 (8)	0.01978 (16)	
S1B	0.38121 (11)	0.51444 (11)	0.21591 (7)	0.02699 (15)	0.6375 (19)
C1B	0.4516 (4)	0.4333 (3)	0.3522 (3)	0.0172 (6)	0.6375 (19)
H1BA	0.4077	0.4784	0.4054	0.021*	0.6375 (19)
C2B	0.5300 (10)	0.3569 (8)	0.1808 (6)	0.0429 (15)	0.6375 (19)
H2BA	0.5390	0.3496	0.1099	0.051*	0.6375 (19)
C3B	0.6279 (10)	0.2492 (8)	0.2775 (7)	0.0299 (7)	0.6375 (19)
H3BA	0.7151	0.1577	0.2789	0.036*	0.6375 (19)
S1X	0.5125 (4)	0.3829 (3)	0.1634 (2)	0.0288 (4)	0.3625 (19)
C1X	0.6323 (13)	0.2441 (10)	0.2839 (10)	0.0172 (6)	0.3625 (19)
H1XA	0.7179	0.1506	0.2871	0.021*	0.3625 (19)
C2X	0.3997 (10)	0.5014 (10)	0.2362 (7)	0.0429 (15)	0.3625 (19)
H2XA	0.3132	0.5990	0.2040	0.051*	0.3625 (19)
C3X	0.4540 (11)	0.4332 (11)	0.3424 (8)	0.048 (2)	0.3625 (19)
H3XA	0.4062	0.4799	0.3943	0.057*	0.3625 (19)
C5B	0.66610 (12)	0.19528 (11)	0.49031 (8)	0.01976 (16)	
H5BA	0.6319	0.2350	0.5472	0.024*	
C6B	0.90351 (12)	0.04953 (11)	0.68276 (8)	0.01932 (16)	
C7B	0.90578 (13)	−0.17204 (11)	0.68809 (8)	0.02196 (17)	
C8B	0.87796 (17)	−0.27885 (13)	0.65086 (10)	0.0302 (2)	
H8BA	0.9231	−0.3832	0.7087	0.045*	
H8BB	0.9279	−0.2790	0.5809	0.045*	
H8BC	0.7649	−0.2436	0.6389	0.045*	
H3NA	0.025 (2)	0.210 (2)	−0.1941 (16)	0.035 (5)*	
H3NB	1.027 (2)	−0.077 (2)	0.8312 (17)	0.037 (5)*	

### Atomic displacement parameters (Å^2^)

**Table:**

	*U*^11^	*U*^22^	*U*^33^	*U*^12^	*U*^13^	*U*^23^
S1A	0.02947 (14)	0.02718 (13)	0.02285 (12)	−0.01128 (11)	−0.00947 (10)	0.00081 (10)
S2A	0.02878 (13)	0.01789 (11)	0.02143 (11)	−0.00851 (9)	−0.00820 (9)	−0.00404 (8)
N1A	0.0232 (4)	0.0199 (3)	0.0164 (3)	−0.0082 (3)	−0.0052 (3)	−0.0033 (3)
N2A	0.0224 (4)	0.0171 (3)	0.0158 (3)	−0.0077 (3)	−0.0046 (3)	−0.0028 (3)
N3A	0.0257 (4)	0.0188 (3)	0.0196 (3)	−0.0092 (3)	−0.0074 (3)	−0.0025 (3)
N4A	0.0279 (4)	0.0187 (4)	0.0213 (4)	−0.0091 (3)	−0.0065 (3)	−0.0029 (3)
C1A	0.0253 (5)	0.0209 (4)	0.0227 (4)	−0.0103 (4)	−0.0057 (3)	−0.0018 (3)
C2A	0.0320 (5)	0.0301 (5)	0.0219 (4)	−0.0149 (4)	−0.0066 (4)	−0.0064 (4)
C3A	0.0295 (5)	0.0233 (4)	0.0187 (4)	−0.0119 (4)	−0.0053 (3)	−0.0044 (3)
C4A	0.0223 (4)	0.0200 (4)	0.0166 (3)	−0.0098 (3)	−0.0041 (3)	−0.0019 (3)
C5A	0.0230 (4)	0.0197 (4)	0.0187 (4)	−0.0088 (3)	−0.0051 (3)	−0.0035 (3)
C6A	0.0209 (4)	0.0187 (4)	0.0160 (3)	−0.0081 (3)	−0.0034 (3)	−0.0034 (3)
C7A	0.0260 (4)	0.0172 (4)	0.0190 (4)	−0.0084 (3)	−0.0043 (3)	−0.0028 (3)
C8A	0.0369 (6)	0.0209 (4)	0.0266 (5)	−0.0126 (4)	−0.0080 (4)	−0.0052 (4)
S2B	0.03028 (13)	0.01731 (11)	0.02452 (12)	−0.00988 (9)	−0.00816 (9)	−0.00420 (9)
N1B	0.0251 (4)	0.0173 (3)	0.0176 (3)	−0.0066 (3)	−0.0066 (3)	−0.0029 (3)
N2B	0.0229 (4)	0.0144 (3)	0.0173 (3)	−0.0064 (3)	−0.0061 (3)	−0.0019 (3)
N3B	0.0275 (4)	0.0197 (4)	0.0183 (3)	−0.0108 (3)	−0.0063 (3)	−0.0028 (3)
N4B	0.0316 (5)	0.0182 (3)	0.0205 (4)	−0.0107 (3)	−0.0081 (3)	−0.0011 (3)
C4B	0.0219 (4)	0.0163 (4)	0.0181 (4)	−0.0083 (3)	−0.0035 (3)	−0.0024 (3)
S1B	0.0283 (3)	0.0208 (2)	0.0227 (2)	−0.00926 (19)	−0.00948 (18)	0.00179 (17)
C1B	0.0168 (9)	0.0083 (7)	0.0140 (7)	0.0010 (6)	−0.0091 (7)	0.0030 (6)
C2B	0.0343 (17)	0.0261 (19)	0.063 (4)	−0.0096 (14)	−0.002 (2)	−0.014 (2)
C3B	0.0346 (15)	0.0358 (15)	0.0226 (16)	−0.0165 (12)	0.0019 (11)	−0.0131 (12)
S1X	0.0313 (8)	0.0265 (9)	0.0240 (5)	−0.0102 (7)	−0.0031 (5)	−0.0066 (5)
C1X	0.0168 (9)	0.0083 (7)	0.0140 (7)	0.0010 (6)	−0.0091 (7)	0.0030 (6)
C2X	0.0343 (17)	0.0261 (19)	0.063 (4)	−0.0096 (14)	−0.002 (2)	−0.014 (2)
C3X	0.053 (4)	0.056 (4)	0.069 (5)	−0.041 (3)	0.029 (3)	−0.044 (4)
C5B	0.0215 (4)	0.0172 (4)	0.0180 (4)	−0.0080 (3)	−0.0034 (3)	−0.0034 (3)
C6B	0.0202 (4)	0.0172 (4)	0.0184 (4)	−0.0075 (3)	−0.0034 (3)	−0.0041 (3)
C7B	0.0268 (4)	0.0152 (4)	0.0190 (4)	−0.0080 (3)	−0.0067 (3)	−0.0010 (3)
C8B	0.0433 (6)	0.0178 (4)	0.0266 (5)	−0.0125 (4)	−0.0124 (4)	−0.0027 (4)

### Geometric parameters (Å, °)

**Table:**

S1A—C1A	1.7085 (11)	N2B—C7B	1.3812 (12)
S1A—C2A	1.7170 (13)	N3B—C6B	1.3430 (12)
S2A—C6A	1.6838 (10)	N3B—N4B	1.3765 (13)
N1A—C5A	1.2859 (13)	N3B—H3NB	0.84 (2)
N1A—N2A	1.3820 (11)	N4B—C7B	1.3063 (13)
N2A—C6A	1.3881 (12)	C4B—C1X	1.381 (13)
N2A—C7A	1.3889 (13)	C4B—C3X	1.392 (10)
N3A—C6A	1.3408 (13)	C4B—C1B	1.410 (3)
N3A—N4A	1.3781 (13)	C4B—C3B	1.429 (8)
N3A—H3NA	0.896 (19)	C4B—C5B	1.4542 (13)
N4A—C7A	1.3026 (13)	S1B—C1B	1.670 (3)
C1A—C4A	1.3787 (15)	S1B—C2B	1.791 (9)
C1A—H1AA	0.9300	C1B—H1BA	0.9300
C2A—C3A	1.3730 (15)	C2B—C3B	1.367 (9)
C2A—H2AA	0.9300	C2B—H2BA	0.9300
C3A—C4A	1.4322 (15)	C3B—H3BA	0.9300
C3A—H3AA	0.9300	S1X—C1X	1.705 (9)
C4A—C5A	1.4568 (13)	S1X—C2X	1.734 (8)
C5A—H5AA	0.9300	C1X—H1XA	0.9300
C7A—C8A	1.4816 (15)	C2X—C3X	1.297 (13)
C8A—H8AA	0.9600	C2X—H2XA	0.9300
C8A—H8AB	0.9600	C3X—H3XA	0.9300
C8A—H8AC	0.9600	C5B—H5BA	0.9300
S2B—C6B	1.6837 (10)	C7B—C8B	1.4809 (15)
N1B—C5B	1.2917 (13)	C8B—H8BA	0.9600
N1B—N2B	1.3977 (11)	C8B—H8BB	0.9600
N2B—C6B	1.3811 (12)	C8B—H8BC	0.9600
			
C1A—S1A—C2A	92.44 (5)	C1X—C4B—C3X	109.6 (5)
C5A—N1A—N2A	120.12 (9)	C1X—C4B—C1B	114.6 (3)
N1A—N2A—C6A	134.03 (8)	C3X—C4B—C3B	107.0 (5)
N1A—N2A—C7A	117.69 (8)	C1B—C4B—C3B	112.0 (3)
C6A—N2A—C7A	108.28 (8)	C1X—C4B—C5B	124.8 (3)
C6A—N3A—N4A	114.25 (8)	C3X—C4B—C5B	125.6 (4)
C6A—N3A—H3NA	127.0 (12)	C1B—C4B—C5B	120.56 (16)
N4A—N3A—H3NA	118.5 (12)	C3B—C4B—C5B	127.5 (3)
C7A—N4A—N3A	104.29 (8)	C1B—S1B—C2B	94.2 (3)
C4A—C1A—S1A	111.67 (8)	C4B—C1B—S1B	111.2 (2)
C4A—C1A—H1AA	124.2	C4B—C1B—H1BA	124.4
S1A—C1A—H1AA	124.2	S1B—C1B—H1BA	124.4
C3A—C2A—S1A	111.42 (8)	C3B—C2B—S1B	107.5 (6)
C3A—C2A—H2AA	124.3	C3B—C2B—H2BA	126.3
S1A—C2A—H2AA	124.3	S1B—C2B—H2BA	126.3
C2A—C3A—C4A	112.42 (10)	C2B—C3B—C4B	115.1 (6)
C2A—C3A—H3AA	123.8	C2B—C3B—H3BA	122.4
C4A—C3A—H3AA	123.8	C4B—C3B—H3BA	122.4
C1A—C4A—C3A	112.04 (9)	C1X—S1X—C2X	91.4 (5)
C1A—C4A—C5A	123.85 (10)	C4B—C1X—S1X	111.6 (5)
C3A—C4A—C5A	124.08 (9)	C4B—C1X—H1XA	124.2
N1A—C5A—C4A	116.66 (9)	S1X—C1X—H1XA	124.2
N1A—C5A—H5AA	121.7	C3X—C2X—S1X	109.7 (7)
C4A—C5A—H5AA	121.7	C3X—C2X—H2XA	125.1
N3A—C6A—N2A	102.63 (8)	S1X—C2X—H2XA	125.1
N3A—C6A—S2A	126.98 (8)	C2X—C3X—C4B	117.6 (8)
N2A—C6A—S2A	130.38 (7)	C2X—C3X—H3XA	121.2
N4A—C7A—N2A	110.54 (9)	C4B—C3X—H3XA	121.2
N4A—C7A—C8A	125.91 (9)	N1B—C5B—C4B	120.21 (9)
N2A—C7A—C8A	123.48 (9)	N1B—C5B—H5BA	119.9
C7A—C8A—H8AA	109.5	C4B—C5B—H5BA	119.9
C7A—C8A—H8AB	109.5	N3B—C6B—N2B	102.76 (8)
H8AA—C8A—H8AB	109.5	N3B—C6B—S2B	127.50 (8)
C7A—C8A—H8AC	109.5	N2B—C6B—S2B	129.69 (7)
H8AA—C8A—H8AC	109.5	N4B—C7B—N2B	110.39 (9)
H8AB—C8A—H8AC	109.5	N4B—C7B—C8B	125.79 (9)
C5B—N1B—N2B	113.61 (9)	N2B—C7B—C8B	123.82 (9)
C6B—N2B—C7B	108.61 (8)	C7B—C8B—H8BA	109.5
C6B—N2B—N1B	128.38 (8)	C7B—C8B—H8BB	109.5
C7B—N2B—N1B	122.26 (8)	H8BA—C8B—H8BB	109.5
C6B—N3B—N4B	113.86 (8)	C7B—C8B—H8BC	109.5
C6B—N3B—H3NB	126.2 (13)	H8BA—C8B—H8BC	109.5
N4B—N3B—H3NB	119.7 (13)	H8BB—C8B—H8BC	109.5
C7B—N4B—N3B	104.32 (8)		
			
C5A—N1A—N2A—C6A	2.18 (18)	S1B—C2B—C3B—C4B	0.9 (10)
C5A—N1A—N2A—C7A	−178.40 (10)	C1X—C4B—C3B—C2B	178 (100)
C6A—N3A—N4A—C7A	0.28 (13)	C3X—C4B—C3B—C2B	−1.7 (9)
C2A—S1A—C1A—C4A	1.06 (10)	C1B—C4B—C3B—C2B	−1.1 (9)
C1A—S1A—C2A—C3A	−0.79 (10)	C5B—C4B—C3B—C2B	178.9 (5)
S1A—C2A—C3A—C4A	0.34 (14)	C3X—C4B—C1X—S1X	0.3 (10)
S1A—C1A—C4A—C3A	−1.04 (13)	C1B—C4B—C1X—S1X	1.0 (10)
S1A—C1A—C4A—C5A	177.06 (9)	C3B—C4B—C1X—S1X	0(19)
C2A—C3A—C4A—C1A	0.45 (15)	C5B—C4B—C1X—S1X	−179.1 (3)
C2A—C3A—C4A—C5A	−177.65 (11)	C2X—S1X—C1X—C4B	0.1 (9)
N2A—N1A—C5A—C4A	179.43 (9)	C1X—S1X—C2X—C3X	−0.6 (9)
C1A—C4A—C5A—N1A	−173.55 (11)	S1X—C2X—C3X—C4B	1.0 (11)
C3A—C4A—C5A—N1A	4.33 (16)	C1X—C4B—C3X—C2X	−0.9 (11)
N4A—N3A—C6A—N2A	−0.02 (12)	C1B—C4B—C3X—C2X	−174 (6)
N4A—N3A—C6A—S2A	179.17 (8)	C3B—C4B—C3X—C2X	−0.9 (10)
N1A—N2A—C6A—N3A	179.21 (11)	C5B—C4B—C3X—C2X	178.6 (6)
C7A—N2A—C6A—N3A	−0.24 (11)	N2B—N1B—C5B—C4B	177.18 (9)
N1A—N2A—C6A—S2A	0.07 (18)	C1X—C4B—C5B—N1B	−3.5 (7)
C7A—N2A—C6A—S2A	−179.39 (9)	C3X—C4B—C5B—N1B	177.1 (5)
N3A—N4A—C7A—N2A	−0.43 (12)	C1B—C4B—C5B—N1B	176.36 (19)
N3A—N4A—C7A—C8A	176.66 (11)	C3B—C4B—C5B—N1B	−3.6 (5)
N1A—N2A—C7A—N4A	−179.11 (9)	N4B—N3B—C6B—N2B	1.89 (12)
C6A—N2A—C7A—N4A	0.44 (13)	N4B—N3B—C6B—S2B	−175.95 (9)
N1A—N2A—C7A—C8A	3.72 (16)	C7B—N2B—C6B—N3B	−2.32 (12)
C6A—N2A—C7A—C8A	−176.73 (11)	N1B—N2B—C6B—N3B	−172.46 (10)
C5B—N1B—N2B—C6B	−49.59 (15)	C7B—N2B—C6B—S2B	175.46 (9)
C5B—N1B—N2B—C7B	141.48 (10)	N1B—N2B—C6B—S2B	5.31 (17)
C6B—N3B—N4B—C7B	−0.69 (13)	N3B—N4B—C7B—N2B	−0.87 (13)
C1X—C4B—C1B—S1B	0.7 (6)	N3B—N4B—C7B—C8B	179.50 (12)
C3X—C4B—C1B—S1B	7(5)	C6B—N2B—C7B—N4B	2.09 (13)
C3B—C4B—C1B—S1B	0.7 (5)	N1B—N2B—C7B—N4B	172.96 (10)
C5B—C4B—C1B—S1B	−179.23 (13)	C6B—N2B—C7B—C8B	−178.27 (11)
C2B—S1B—C1B—C4B	−0.2 (4)	N1B—N2B—C7B—C8B	−7.40 (17)
C1B—S1B—C2B—C3B	−0.4 (7)		

### Hydrogen-bond geometry (Å, °)

**Table:**

*D*—H···*A*	*D*—H	H···*A*	*D*···*A*	*D*—H···*A*
N3A—H3NA···S2B^i^	0.894 (19)	2.46 (2)	3.3494 (11)	177.7 (18)
N3B—H3NB···S2A^ii^	0.84 (2)	2.45 (2)	3.2728 (12)	167.7 (19)
C5A—H5AA···S2A	0.93	2.50	3.2311 (12)	135
C8B—H8BA···N4A^iii^	0.96	2.59	3.5503 (16)	175
C5B—H5BA···Cg1^iv^	0.93	2.91	3.4955 (12)	122

Symmetry codes: (i) *x*−1, *y*, *z*−1; (ii) *x*+1, *y*, *z*+1; (iii) *x*+1, *y*−1, *z*+1; (iv) −*x*+1, −*y*, −*z*+1.
